# Spina bifida, the normal, the pathological and the in-between: first evidence from a forensic osteological collection

**DOI:** 10.1007/s00414-023-03066-2

**Published:** 2023-07-31

**Authors:** Maria Torres Manso, Vitor M. J. Matos

**Affiliations:** 1 Department of Life Sciences, University of Coimbra, Calçada Martim de Freitas, 3000-456 Coimbra, Portugal; 2 Research Centre for Anthropology and Health, Department of Life Sciences, University of Coimbra, Calçada Martim de Freitas, 3000-456 Coimbra, Portugal

**Keywords:** Sacrum, Spina Bifida, Cleft Neural Arch, Forensic Anthropology Population Data, Portugal

## Abstract

**Abstract:**

Spina bifida (SB), a rare congenital disorder, is often mentioned as an individualizing factor in Forensic Anthropology. A lack of empirical data regarding SB is noticed in the scientific literature. Moreover, within the scope of anthropological research on SB disparities in terminology, classification systems, and methodological approaches result in incomparable results. The wide range (1,2%-50%) of “spina bifida occulta” reported prevalences is a good example. This research aims to analyze and debate the standard diagnostic criteria of SB on human skeletal remains, and attempts to elaborate on an universal system, premised on the distinction between SB as a pathology, and cleft neural arch (CNA) as an anatomical variant, according to Barnes (1994, p. 360 [1). A study-base of 209 individuals (88 males; 121 females; 44–99 years old) from the *21st Century Identified Skeletal Collection* from the University of Coimbra (CEI/XXI) was macroscopically analyzed, focusing on the sacrum and remaining vertebrae. Four individuals presented complete posterior opening of the sacral canal (2,6%[4/156]). The observed bone changes, combined with the analysis of the entire skeleton, indicate that CNA, rather than SB linked to a neural tube defect, is the most reliable explanation for these cases. Overall, CNA was observed on 11 skeletons (7.05% of 156). The viability and applicability of the developed methodology for the identification of SB/CNA in forensic and/or osteological contexts are discussed, as well as the possibility of a lower prevalence of SB occulta, in the general population, than speculated before.

**Highlights:**

• Spina bifida has been studied so far under different methodologies, classification systems and nomenclature, leading to unstandardized and incomparable data.

• Spina bifida as a pathological manifestation of a neural tube defect, as opposed to cleft neural arch as a simple form of skeletal variation.

• Both spina bifida and complete sacral cleft fit the criteria of an individualizing trait in Forensic Anthropology.

**Supplementary Information:**

The online version contains supplementary material available at 10.1007/s00414-023-03066-2.

## Introduction

Personal identification has always been an important issue in human societies, particularly the identification of human remains, which has gained even greater relevance in recent times [2]. In 1996, the universal right to be identified after death was recognized by the General Assembly of INTERPOL [3].

In the processes of identifying human remains there are a number of requirements, both biological and technical, that a scientific method must fulfill, to be considered valid, such as being based on characteristics that are “unique and immutable” (2, p. 401). At the same time, scientific methods must be testable, replicable, reliable, and scientifically valid [4, 5].

Forensic Anthropology (FA) contributes to the identification process by analyzing the biological profile, post mortem interval, traumatic injuries, aspects of the circumstances of death and individualization factors [4, 6]. These are characteristics that last during decomposition and are recognizable after death, representing permanent changes in the morphology of the skeleton and reflecting events in the individual’s life, such as cultural modifications, trauma, surgical interventions or pathological conditions [7, 8]. For a morphological feature to be considered an individualization factor, it has to occur with a low enough frequency (< 10%), and it has to be easily observable in imaging exams and dry bone [9, 10]. The value of these characteristics also depends on the existence of medical records, such as ante mortem radiographs, which allow comparison with post mortem observations. Distinguishing between simple anatomical variation and pathological change requires an in-depth knowledge of osteology and extensive experience in observing skeletal variation and common anomalies [9, 10].

The spectrum of skeletal manifestations associated with Spina bifida (SB) is a good example of the challenges when distinguishing between normal and pathological in dry bone. SB is a congenital disease that consists of the incomplete fusion of the vertebral arches, associated with a failure in the development of the neural canal [11]. It can occur in one or more vertebrae and in any region of the spine, more commonly in the lumbar and sacrum. Like other congenital diseases, the etiology of SB is multifactorial, involving the interaction of genetic and environmental factors [12]. Several studies have found evidence that SB is associated with a maternal folic acid deficiency during the embryonic period [13, 14].

The neural tube is the brain’s precursor to the spinal cord, which during the embryonic period develops through a process called neurulation, that is divided into a primary and secondary phase [12]. Defects that occur during primary neurulation result in open lesions, that is, they are not covered by normal skin [1]. Anomalies that may result from this stage of development are anencephaly (the absence of a brain) and meningomyelocele, or in rarer cases myelocele. In addition to the neurological deficiencies that arise in the spinal cord, meningomyelocele is accompanied by SB cystica that manifests in vertebrae with thin pedicles, deformed and/or absent laminae, underdeveloped spinous apophyses, and the edges of the bone anomaly are elevated by the protruding cystic mass [1].

When defects occur during the secondary neurulation, the resulting lesions are closed, meaning they are covered by normal skin, and are accompanied by SB occulta, as is the case with meningocele. This anomaly occurs most frequently in the lumbosacral region. The associated symptoms vary depending on the expression of the lesion [1].

The presence of cleft vertebral segments may not be associated with a neural tube defect (NTD), as the other conditions mentioned so far. This failure of the vertebral arch to fuse without the occurrence of neural tube defects (NTDs) is often considered a simple anatomical variant, which occurs mostly in the sacrum, without any clinical implications and is more common than cases involving NTDs [1].

It is important to emphasize that there is great divergence in the literature regarding how NTDs and SB are defined and classified. This discrepancy, both in the theoretical framework and in the practical context, is a problem recognized by several researchers [12, 15–19]. Differences in the nomenclature used, classification systems and methods of analysis lead to incomparable results, as illustrated by the very high variation in the prevalence of SB occulta obtained in different population studies (between 1.2% and 50%) [15, 16]. An author who sought to establish a method to standardize the analysis of SB was Barnes [1] by separating the anomaly that occurs in the neural tube and the bone lesion that is observed in the spine, considering these two phenomena as distinct, but intrinsically associated. NTDs such as meningomyelocele or meningocele are congenital anomalies that occur in the nervous system while SB refers to a lesion that occurs in the skeletal system. The occurrence of SB may be a consequence of a NTD, which in Barnes’s view is the case of both SB cystica and occulta, it may also be an isolated phenomenon. In this case, the author suggests the name of “Cleft Neural Arch” (CNA). This condition usually affects only one or two vertebral segments in the transition regions between different types of vertebrae, and in certain cases it may extend to the entire dorsal sacral wall. Mulhern and Wilczak [18] also chose a similar terminology, “Complete Cleft Sacra” instead of SB, to specifically describe the condition they observed in skeletal remains from Native Americans.

The terminological disparity exposed here is reflected in a central point of discussion within the topic of SB, which is the debate on the clinical importance of SB occulta. Many authors do not distinguish between SB occulta and CNA using the first formulation to refer to both [12, 17, 19]. However, SB occulta has associated symptomatology and therefore clinical importance, whereas CNA does not [1].

Identifying the presence or absence of a NTD and therefore SB through the observation of dry bone is very difficult or even impossible. Both NTDs and the CNA frequently occur in the lumbosacral region, and both can result in a completely open sacrum [1]. In NTDs the medullary canal is widened, pushing the edges of the bony cleft outwards, comparatively, when there is no NTD the medullary canal remains open, but it is not enlarged and the edges are not raised [1].

Paleopathological studies mainly analyze SB occulta in the sacrum, because, in general, in an archaeological context, when SB is found in an adult skeleton, it can be assumed that it was not a case of SB cystica, due to the low life expectancy associated with it [17]. Still, there are reported paleopathological cases of SB cystica. At the Windover archaeological site (Florida, USA), an individual aged 15 years old was found with pathological changes possibly associated with SB cystica [20]. Garralda et al. [21] identified an opening in the L5 vertebral arches and sacral vertebrae in a child aged 8–9 years old, from a necropolis in Cathedral of El Burgo de Osma cloister (Soria, Spain). This lesion, together with the presence of hydrocephalus led to the diagnosis of SB cystica with meningomyelocele. These cases demonstrate the importance of a detailed analysis of the entire skeleton and differential diagnosis as essential tools to final conclusions. The presence of other skeletal anomalies beyond those occurring at the sacrum can contribute to the most correct classification of the sacral lesions observed [17].

The relevance of SB to FA and its value as an individualizing factor cannot really be ascertained within the current theoretical framework, as no method of classifying this pathology can fit the aforementioned criteria, to be considered valid, while there isn’t an agreement amongst researchers, in the clinical and paleopathological domains, on its definition and classification. The following cases exemplify this well. In a study on the relevance of discrete traits for FA, Verna and co-authors [10] found eight traits having a prevalence lower than 5%, including SB occulta. The authors consider SB occulta to be an asymptomatic form resulting from an ossification failure during the posterior arch fusion. In another similar research, which investigates whether certain morphological characteristics are rare enough to constitute individualization factors, Komar and Lathrop [8] consider that SB occulta has minimal value as an individualizing characteristic, since it would not be detectable during life, by the individual or his family. The authors do not specify what they consider to be SB occulta, but refer to it as an anomaly, which suggests that they define it similarly to Verna et al. [10]. Therefore, it is important to establish a classification system/method approved and applied by the entire scientific community, followed by a review of past studies to standardize the existing data.

The main goals of this research were to highlight the existing divergences in the literature regarding SB, propose a system that allows the analysis of SB in an objective and replicable manner and apply it in the 21^st^ Century Identified Skeletal Collection of the University of Coimbra, arguing the relevance of this condition in FA. To our knowledge, this is the first systematic work of this kind, in an identified collection with forensic relevance.

## Material and methods

A sample derived from the 21^st^ Century Identified Skeletal Collection from the University of Coimbra (CEI/XXI) was studied [22]. Housed at the Laboratory of Forensic Anthropology (LFA) of the Department of Life Sciences at the University of Coimbra, Portugal, the skeletons from CEI/XXI originate from the Capuchos Cemetery in Santarém, Portugal [23, 24]. It is currently composed of 302 adult individuals of both sexes (162 females and 140 males), who died between 1982 and 2012 and were exhumed between 1999 and 2016. The ages at death range from 25 to 101 years old [24].

The studied sample comprised 209 skeletons. The proportion of females (57.9%; *n* = 121) is significantly higher (χ^2^ = 5.211; d.f. = 1; *p* = 0.022) than that of males (42.1%; *n* = 88). The 207 individuals with age at death information were divided into six age groups (Table 1), which ranged between 44–99 years (mean = 80.73; median = 82; SD = 10.059).

**Table 1:** Sample distribution by sex and age groups

			Age Groups (years)						Total
			40–49	50–59	60–69	70–79	80–89	90–99	
Sex	Female	Frequency	0	1	12	23	55	29	120
		% within Sex	0,0%	0,8%	10,0%	19,2%	45,8%	24,2%	100,0%
		% within Age Groups	0,0%	33,3%	40,0%	43,4%	67,9%	74,4%	58,0%
	Male	Frequency	1	2	18	30	26	10	87
		% within Sex	1,1%	2,3%	20,7%	34,5%	29,9%	11,5%	100,0%
		% within Age Groups	100,0%	66,7%	60,0%	56,6%	32,1%	25,6%	42,0%
Total		Frequency	1	3	30	53	81	39	207
		%	0,5%	1,4%	14,5%	25,6%	39,1%	18,8%	100,0%

In addition, a set of 31 sacra (training sample) from the Unidentified Osteological Collections of the Department of Life Sciences of the University of Coimbra (see Bárrios, [25]) were randomly selected for training purposes. From this sample, the parameters to be applied in the CEI/XXI were developed and the inter- and intra-observer errors were calculated using Cohen’s Kappa. ﻿

The sacra were macroscopically analyzed considering the state of preservation according to Buikstra and Ubelaker [26] (>75%, complete; 25% – 75%, partially complete; <25%, incomplete), the number of *foramina* and the opening of the median sacral crest, both proximally and distally. The remaining vertebrae were inspected for gross morphological changes, especially those located at the posterior vertebral (neural) arch.

The exact anatomical proximal and distal limits of the median sacral crest were determined having as topographical references the associated *foramina* pairs and the respective sacral vertebrae involved. In those individuals who presented complete opening of the sacral canal, a more detailed morphological analysis was done, in order to differentiate between SB cystica, SB occulta and CNA, based on criteria proposed by Barnes [1]. According to this author, the manifestation of SB cystica associated with meningomyelocele usually involves several vertebrae and occurs mostly in the lumbosacral region. In the unfused vertebral arches, the pedicles are thin, the laminae are deformed or absent, the spinous process does not develop, and the edges of the skeletal defect are raised by the cyst. Similarly, SB occulta associated with meningocele occurs predominantly in the lumbosacral region. It results in pedicles and vertebrae with a wider appearance and is distinguished from SB cystica by manifesting in a smaller and narrower lesion that may be fusiform when several vertebrae are affected. Finally, CNA is characterized by not being associated with a NTD, unlike the conditions mentioned above. Thus, the edges of the bony defect are not raised, and the spinal canal remains normal. It usually involves one or two vertebral segments, in the transition regions between different types of vertebrae, but it can also cause fusion failure of the sacral dorsal wall [1]. As SBC and SBO occur in association with a NTD, these are accompanied by other neurological manifestations in the rest of the body, namely different levels of paralysis depending on the site and extent of the neurological defect, gait disturbances and foot deformities, among others [1]. Therefore, the complete skeleton of these individuals was analyzed in order to check for evidence of additional bone changes that could be associated with the observed defect in the sacrum towards a differential diagnosis, if needed. The data collected was systematically recorded (Excel, Microsoft) and subjected to a statistical study (IBM/SPSS Statistics version 21.0). Expected and observed frequencies in the categories under analysis were evaluated using Chi-square test (χ^2^), including Yates corrected Chi-square in the case of 2 × 2 contingency tables and Fisher’s exact test when Chi-square basic assumptions were not fulfilled. Correlation analyses were performed using Spearman’s coefficient. Binary logistic regression was used, with *p* values ​​determined by Wald’s chi-square test [27]. Statistical significance was considered for *p* < 0.05.

The inter- and intraobserver errors were calculated using Cohen’s Kappa (Table S1). In general, there were no noticeable discrepancies, either in the inter- and intraobserver errors, except for the variable proximal point of the sacral crest (Table S1). In this variable, the differences between observations arise because one observer (MTM) considered the proximal origin of the sacral crest between two vertebrae, while observer two (VMJM) had the sacral vertebra as reference and not the intervertebral spaces.

## Results

**Fig. 1:**
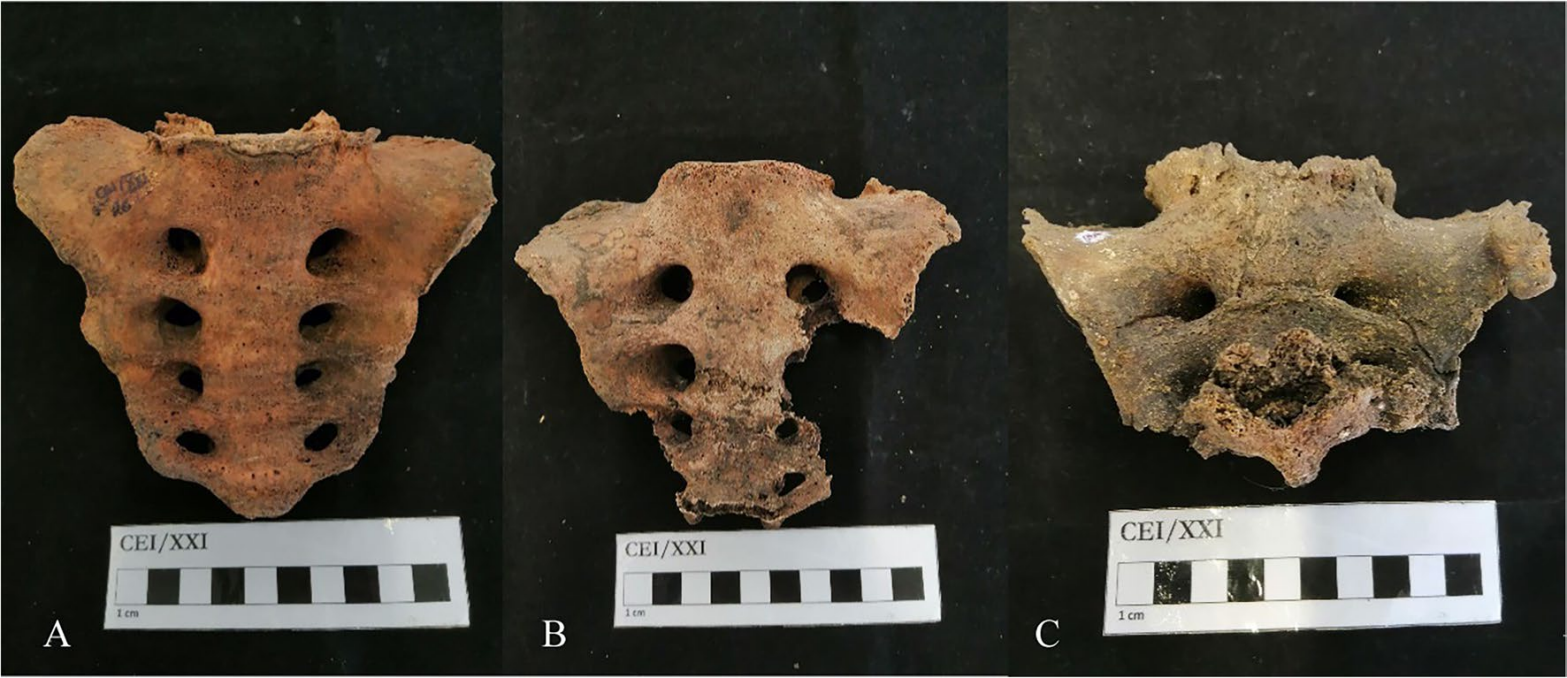
Different states of preservation: **A** Subject CEI/XXI_26, complete sacrum; **B** Subject CEI/XXI_182, partially complete sacrum; **C** Subject CEI/XXI_153, incomplete sacrum

The 209 observed sacra were complete (52.2%, *n* = 109), partially complete (27.3%, *n* = 57) or incomplete (20.6%, n = 43). Figure 1 illustrates the different preservation states found in the CEI/XXI.

The observation of the most proximal point of the sacral crest, which corresponds to the location where it starts, was possible in 181 of the 209 sacra under study. This point varied between S1 (71.3%[129/181]) – or L5 (5.5%[10/181]) in those cases presenting sacralization of the last lumbar vertebra – and S3 (0.6%[1/181]). The sacral crest started at the S2 level in six sacra (3.3% of 181). This wide amplitude demonstrates the great variability of this sacral anatomical element.

Distally, the opening of the sacral canal comprised the S4 and S5 vertebrae in most individuals (71.2%[111/156]). This is usually known as the sacral hiatus, which in some individuals also included the S3 (9.0%[14/156]) and in others was limited to the S5 (17.3%[27/156]).

**Fig. 2:**
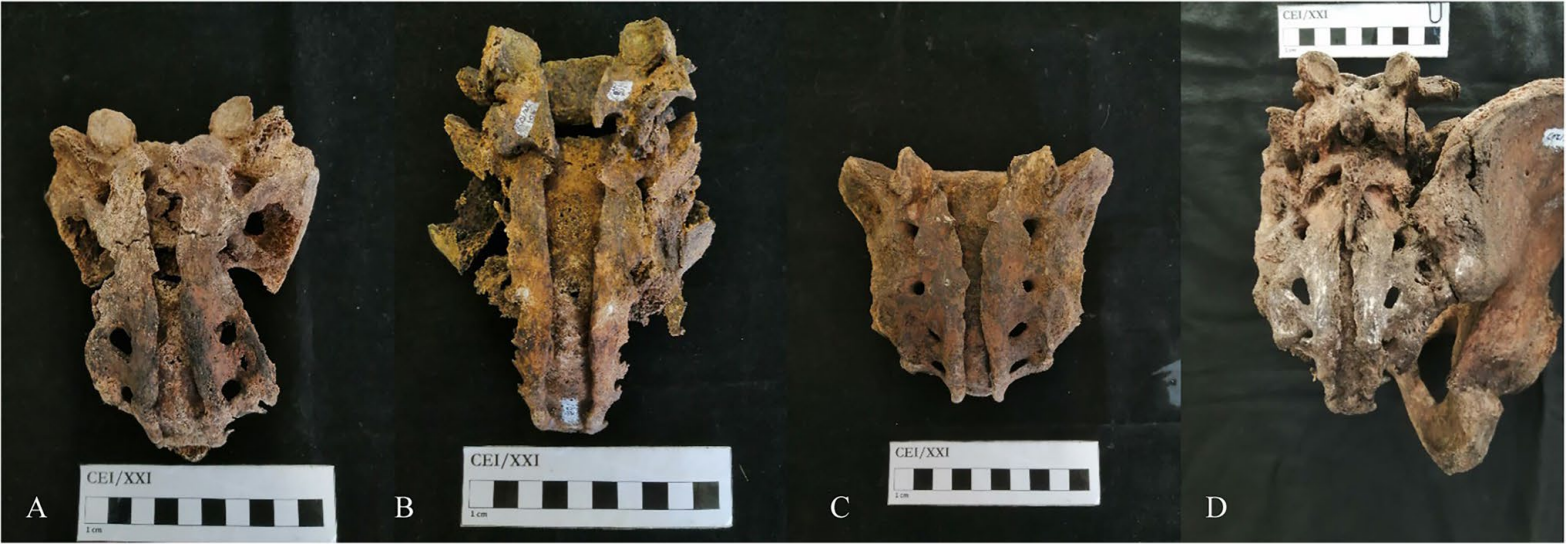
Complete sacral cleft: **A** Subject CEI/XXI_79; **B** Subject CEI/XXI_106, in this case there was also a cleft in the neural arch of L5; **C** Subject CEI/XXI_185; **D** Subject CEI/XXI_293

The complete opening of the sacral canal was detected in only 4 individuals (2.6%[4/156]), and in one of these (CEI/XXI_106) the opening of the posterior arches extends to L5 (see Fig. 2). Of these 4 individuals three are men (75%[3/4]) (Table 2). Although the prevalence recorded in males (4.4%[3/68]) is higher than in females (1.1%[1/88]) (Table 2), no significant differences between sexes were found (Fisher exact test: *p* = 0.318). Each individual, of those with complete opening of the sacral canal, belongs to a different age group, as Table 3 shows with the lowest age at death being 65 years old (CEI/XXI_185) and the highest 90 years old (CEI/XXI_106).

**Table 2:** Distribution of the observed sacra by opening of the sacral canal (dichotomized variable) and sex

			Sex		Total
			Female	Male	
Opening of the sacral canal (dichotomized variable)	Open sacral canal	Frequency	1	3	4
		% within sex	1,1%	4,4%	2,6%
		% within opening of sacral canal	25,0%	75,0%	100,0%
	Closed sacral canal	Frequency	87	65	152
		% within sex	98,9%	95,6%	97,4%
		% within opening of sacral canal	57,2%	42,8%	100,0%
Total		Frequency	88	68	156
		%	56,4%	43,6%	100,0%

**Table 3:** Distribution of the observed sacra by opening of the sacral canal (dichotomized variable) and age groups

			Age groups						Total
			40–49	50–59	60–69	70–79	80–89	90–99	
Opening of the sacral canal (dichotomized)	Open sacral canal	Frequency	0	0	1	1	1	1	4
		% within opening of sacral canal	0,0%	0,0%	25,0%	25,0%	25,0%	25,0%	100,0%
		% within age groups	0,0%	0,0%	4,5%	2,1%	1,7%	4,2%	2,6%
	Closed sacral canal	Frequency	1	3	21	46	57	23	151
		% within opening of sacral canal	0,7%	2,0%	13,9%	30,5%	37,7%	15,2%	100,0%
		% within age groups	100,0%	100,0%	95,5%	97,9%	98,3%	95,8%	97,4%
Total		Frequency	1	3	22	47	58	24	155
		%	0,6%	1,9%	14,2%	30,3%	37,4%	15,5%	100,0%

The applied binary logistic regression model (see Table S2) shows that the independent variables, namely sex (χ2 Wald = 1.494; *p* = 0.222) and age at death (χ2 Wald = 0.050; *p* = 0.823), are not significant predictive factors for the complete opening of the sacral canal (dependent variable).

When crossing the data obtained in the analysis of the most proximal point of the sacral crest with those of the opening of the sacral canal, it was verified that, in addition to the four individuals mentioned above, two others have very reduced sacral crests, which extend for only one or two vertebral segments (Table 4). In particular, individuals CEI/XXI_126 (male; 78 years old) and CEI/XXI_265 (female; 79 years old), whose sacral crests cover only vertebrae S3 and S4 and S2 and S3, respectively. In these individuals, there is, in fact, a fusion of the vertebral arches and, at the same time, the lack of it, both proximally and distally.

**Table 4:** Cross analysis of data obtained from the opening of the sacral canal with the proximal point of the median sacral crest

			Proximal point of median sacral crest						Total
			L5	S1	between S1 & S2	S2	S3	Absent crest	
Opening of the sacral canal	S1-S5	Frequency	0	0	0	0	0	4	4
		% within opening of sacral canal	0,0%	0,0%	0,0%	0,0%	0,0%	100,0%	100,0%
		% within proximal point of crest	0,0%	0,0%	0,0%	0,0%	0,0%	100,0%	2,6%
	S3-S5	Frequency	0	9	4	1	0	0	14
		% within opening of sacral canal	0,0%	64,3%	28,6%	7,1%	0,0%	0,0%	100,0%
		% within proximal point of crest	0,0%	8,1%	15,4%	16,7%	0,0%	0,0%	9,0%
	S4-S5	Frequency	8	80	17	5	1	0	111
		% within opening of sacral canal	7,2%	72,1%	15,3%	4,5%	0,9%	0,0%	100,0%
		% within proximal point of crest	100,0%	72,1%	65,4%	83,3%	100,0%	0,0%	71,2%
	S5	Frequency	0	22	5	0	0	0	27
		% within opening of sacral canal	0,0%	81,5%	18,5%	0,0%	0,0%	0,0%	100,0%
		% within proximal point of crest	0,0%	19,8%	19,2%	0,0%	0,0%	0,0%	17,3%
Total		Frequency	8	111	26	6	1	4	156
		%	5,1%	71,2%	16,7%	3,8%	0,6%	2,6%	100,0%

## Discussion

### Sacral crest and sacral cleft

The analysis of the sacral crest, both proximally and distally, in the 209 skeletons from the CEI/XX provided new evidence regarding the anatomical variation and pathological manifestations affecting this bone. By determining at what segment the sacral crest starts proximally, it is possible to establish whether there has been a failure in the fusion of the vertebral arches of S1 and S2, which is often considered as SB occulta and to which clinical importance is attributed [12, 16, 28, 29].

Fidas et al. [29] analyzed the L5, S1 and S2 vertebrae, in a sample of radiographs from 2707 individuals from Scotland (1359 men and 1348 women), in order to evaluate the presence or absence of an opening of the vertebral arches, as well as its morphology. They found a cleft in the arch of the S1 in 397 individuals (14.7%) and of S1 and S2 in 166 (6.13%), which are values ​​considerably higher than those obtained in the present study (S1 in 3.3% and S1 + S2 in 0.55% out of 181). It is important to underline the different age profile of the sample from Fidas and co-authors, with more individuals in younger age groups. Several authors have reported higher prevalence of defects related to SB or failure of vertebral arch fusion in younger age groups, which decrease with age [16, 18, 28, 29]. This decrease is related to a continuous ossification at the opening of the vertebral arches, linked to the common degenerative process that happens with advancing age [29]. When comparing data from different studies the underlying methodological approaches—plain X-ray vs dry bone analysis—associated biases must be considered. For example, Albrecht and colleagues [15] suggest an underestimation of sacral fusion through radiological analysis. Molto and colleagues [12] also examined the opening at all vertebral levels of the sacrum individually, in an archaeological sample from three cemeteries in the Dakhleh oasis in Egypt. They reported failure of vertebral arch fusion in 10.4% (46/442) at S1 level and in 2.3% (10/442) at S1 to S2 level.

The sacral hiatus, which is a normal feature of almost all sacra, corresponds to the opening of the sacral canal in the most distal part of the sacrum [30]. In 71.2% (111/156) of our study-base, the sacral hiatus comprises the S4 and S5 vertebrae. Additionally, it was extended to the S3 level in 9.0% (14/156) and was limited only to S5 in 17.3% (27 /156) of the sacra.

In two Indian samples, one with 270 sacra [31] and the other with 75 sacra [32], the beginning of the sacral hiatus was found to be at the S4 level in 55.9% (147/263) and 48.0% (32/75) of the individuals, respectively. In both samples, a high percentage of sacra had the sacral hiatus from S3 onwards, respectively 37.3% (98/263) and 43.6% (32/75). On the contrary, in both samples few cases of sacral hiatus limited to the S5 were found, respectively 3.4% (9/263) and 5.3% (4/75) [31, 32]. Another study, based on 92 sacra of Japanese individuals, presents results more similar to those obtained in the present study, with the sacral hiatus starting at S4 with a prevalence of 65.2% (60/92), starting at S3 in 14.1% (13/92) and 15.2% (14/92) in S5 [33].

As for the complete opening of the sacral canal, the total prevalence obtained in the present study was 2.6% (4/156), 4.4% (3/68) in males and 1.1% (1/88) in females, a non-significant difference between sexes. These values ​​are consistent with the results presented in the literature. In the Hamann-Todd Osteological Collection (Cleveland, USA), Eubanks and Chevuru [16], observed this condition in 1.2% (35/2866) of the individuals studied, and it was more common in men and also in individuals of European ancestry ("whites”). In a sample of Amerindians, Mulhern and Wilczak [18] recorded the occurrence of complete opening of the sacral canal in 32 sacra in a total of 1943 subjects (1.6%), comprising 20 cases in 965 men (2.1%), 9 in 852 women (1.1%) and 3 in 126 individuals of unknown sex. The differences between the sexes were not significant. In the same study with the aforementioned Egyptian sample [12], 12 subjects (2.7% of 442) exhibited complete opening of the sacral canal, of which two were female (0.8% of 252) and 10 were male (5.2% of 190), a difference that was statistically significant. In the *Coleção de Esqueletos Identificados* (CEI) of the University of Coimbra (nineteenth-twentieth centuries) the complete absence of the medial sacral crest was observed in 10 of the 456 skeletons studied, corresponding to a prevalence of 2.19%. As in other studies, men were more affected (3.18%[8/251]) than women (0.98%[2/205]), although there were no statistically significant differences [34].

### Spina Bifida as a pathology

The criteria chosen for the macroscopic analysis of the osteological material allowed an objective and complete observation of the sacrum and its variability, without attending to previously defined classifications. As such, the collected data can be confronted and discussed in the light of different studies developed in the scope of SB, regardless of whether these correspond to the criteria adopted throughout this study for the presence of SB, as a pathological alteration. Through in-depth bibliographic research and considering the theoretical constraints previously exposed, we sought to outline criteria for the diagnosis of SB through the analysis of skeletal remains.

A main reason why existing data on SB is so inconsistent and disparate is the wide range of anomalies or defects that are considered to be SB occulta, ranging from small variations, such as failure in the fusion of an isolated vertebral arch, to more expressive lesions. Fidas et al. [29] consider SB occulta in vertebrae L5, S1 and S2, either alone or together. According to these authors, the presence of “spina bifida occulta”, or in other words, the cleft of the posterior arches of these vertebrae is closely associated with neurological problems in the sacral segments of the spinal cord, including urological problems. They argue that considering that these are common problems, its clinical importance should not be disregarded [29]. Avrahami and coauthors [28] adopt a similar position, arguing that “S1 spina bifida occulta is not an innocent finding”, which is linked to a (statistically significant) higher incidence of herniated discs between L4 and L5 or L5 and S1, in both sexes and all ages. It is important to mention that the possibility of SB occulta occurring from L5 to S2, or in only one of these vertebrae, cannot be ruled out, and if that is the case, the morphology of the defect will be a determining factor for the final diagnosis. Despite the potential clinical importance of vertebral arch cleft of an isolated vertebra, especially in the lumbosacral region, these variations were not classified as SB occulta in the course of the present study. Although individuals with opening of the sacral canal have become the focus of study, SB does not affect only the sacrum and can occur in other vertebrae, especially in the lumbar region.

According to the criteria assumed here, the detection of the failure in the fusion of the vertebral arches does not necessarily mean the presence of SB. This is because, it is considered that SB is the manifestation, in the skeletal system, of the neural tube defect (nervous system) that occurs during embryonic development. Therefore, the failure in the fusion of the vertebral arches does not imply that there was NTD, thus being a case of CNA, which is a bone malformation without neurological involvement. As such, after observing that there is a failure in the fusion of the posterior arches, one should examine the morphology of the bone defect to try to distinguish between the different conditions. In our study, the individuals whose bone defect could potentially be SB were only the four with complete opening of the sacral canal, however the analysis suggests that the observed changes are more compatible with a CNA. Kumar and Tubbs [17] argue that it is not possible to distinguish between NTDs, and between their presence or absence, through the analysis of skeletal remains, as the manifestations in the vertebrae and potential neurological consequences of the different conditions are too similar. Although no individual in this sample was identified as having SB or an NTD, it is thought that the distinction between the presence of these and a CNA is possible, based on the information present in the literature. It is also hypothesized that the distinction between types of SB or NTD is superfluous, or even impossible in dry bone. The exercise of a differential diagnosis should always be part of this process. In addition to Barnes [1], whose work served as the basis for this research, Mulhern and Wilczak [18] allude to this distinction, highlighting a single sacrum in their sample whose morphology of the opening of the sacral canal is clearly morphologically different from the others analyzed. The authors do not present a specific diagnosis but note the difference and discuss the hypothesis that the individual had suffered from an NTD or spinal dysraphism [18]. Kumar and Tubbs [17] developed an extremely detailed exposition of the complexity of this debate, which should be better explored in future works, in osteological material, with the complement of more in-depth knowledge of embryology and the clinical context of SB and NTDs.

The prevalence of “spina bifida occulta” reported in different studies varies between 1.2% and 50% [16]. The consideration of SB or CNA as a narrower range of conditions led to a mitigation of such a wide variation in prevalence. The results obtained, in combination with data from other studies, whose methodology and structure allowed the comparison, reveal a variation in the prevalence of opening of the sacral canal between 1.2% and 2.7%. Furthermore, CNA was observed in a total of 11 subjects (7.05%[11/156]), that is, in the 4 subjects with complete opening of the sacral canal, plus 6 others with vertebral arch cleft of S1 and one with cleft in both S1 and S2. This malformation is presumed to have a much greater prevalence than complete sacral cleft which is a form of CNA. SB itself is an even rarer condition.

Both complete sacral cleft and SB can be considered as individualizing factors in the context of personal identification and FA. The value each one brings to the identification process will always depend on the existence of ante mortem records and will differ, since SB has variable clinical consequences in the lives of individuals and that CNA is mostly a silent condition. Despite the relevance, in a forensic context of the anatomical variation and congenital anomalies discussed in this article, these aren’t generally considered in published forensic case reports. Their importance is thus relative to whether and individual with these conditions is found which is a rare occurrence. However, it is argued that by considering SB as a whole, and the consequence of a neural tube defect, therefore distinguishing it from neural arch cleft, a simple form of variation with no impact in the individual’s life, as opposed to the way it is most commonly identified in paleopathological literature, allows to narrow the diagnosis and increase its value as an identification factor. An individual with SB occulta, as it is defined throughout this article, is likely to have ante mortem imagiological scans as it would be something that majorly affected their health and daily life, also it would most likely have been diagnosed at birth. This diagnosis would not lead to a positive identification, but as many anthropological methods in forensics and personal identification, it may help narrow down the possible matches, or lead to an exclusion.

## Conclusion

This is the first systematic study of SB and CNA in an identified collection relevant to FA. Complete sacral cleft was found in four individuals (2.6%) from the CEI/XXI. Although there were no significant differences between sexes, the statistical study showed that men are four times more likely to have complete sacral cleft. The presence of SB was not found in any of the individuals, and the bony defect observed in each of them was considered to be a CNA. Altogether in the sample, 11 individuals had CNA (7.05%).

Therefore, instead of considering a range of variation in the prevalence of “spina bifida occulta” between 1.2% and 50%, as reported in the literature, we sought to compare the prevalence of complete sacral cleft with studies whose results were compatible, leading to a variation between 1.2% and 2.7%. SB itself is even rarer. Thus, both complete sacral cleft and the SB can be considered as individualizing factors in the context of personal identification and FA.

The continuation of the research of SB within the context of FA and paleopathology should focus on the standardization of methodologies and classification systems applied, mainly with regard to the distinction between the presence of SB occulta or CNA, in order to develop compatible and realistic data about their prevalence.

### Supplementary Information

Below is the link to the electronic supplementary material.

Supplementary file1 (DOCX 19 KB)

## Data Availability

The data collected is available under request.
