# Supplementary material for: Spina bifida, the normal, the pathological and the in-between: first evidence from a forensic osteological collection
Source: Int J Legal Med. 2023 Jul 31;138(1):249–58. doi: 10.1007/s00414-023-03066-2 (PMC10771999; doi:10.1007/s00414-023-03066-2)
Supplement: Supplementary file 1 — Supplementary file1 (DOCX 19 KB) [file 414_2023_3066_MOESM1_ESM.docx]

**Supplemental Materials**

Table S1- Cohen's Kappa results for inter and intraobserver error.

|  | | | intraobserver error | interobserver error |
| --- | --- | --- | --- | --- |
| Median crest (proximal point) | Cohen's Kappa | Cohen's Kappa value | 0,628 | 0,419 |
|  |  | *p* | > 0,001 | > 0,001 |
| Opening of sacral canal | Cohen's Kappa | Cohen's Kappa value | 0,848 | 0,848 |
|  |  | *p* | > 0,001 | > 0,001 |

Table S2- Results from the binary logistic regression analysis for the opening of the sacral canal considering sex and age as independent variables (adapted from the output from IBM SPSS Statistics, version 21.0).

| **Variables in the Equation** | | | | | | | |
| --- | --- | --- | --- | --- | --- | --- | --- |
|  | | B | S.E. | Wald | df | Sig. | Exp(B) |
| Step 1^a^ | Sex(1) | 1,476 | 1,208 | 1,494 | 1 | ,222 | 4,376 |
|  | Age | -,012 | ,055 | ,050 | 1 | ,823 | ,988 |
|  | Constant | 4,002 | 4,276 | ,876 | 1 | ,349 | 54,702 |
| a. Variable(s) entered on step 1: Sex, Age. | | | | | | | |
